# How Listeners Weight Acoustic Cues to Intonational Phrase Boundaries

**DOI:** 10.1371/journal.pone.0102166

**Published:** 2014-07-14

**Authors:** Xiaohong Yang, Xiangrong Shen, Weijun Li, Yufang Yang

**Affiliations:** 1 Key Laboratory of Behavioral Science, Institute of Psychology, Chinese Academy of Sciences, Beijing, China; 2 College of Humanities and Communications, Shanghai Normal University, Shanghai, China; UNLV, United States of America

## Abstract

The presence of an intonational phrase boundary is often marked by three major acoustic cues: pause, final lengthening, and pitch reset. The present study investigates how these three acoustic cues are weighted in the perception of intonational phrase boundaries in two experiments. Sentences that contained two intonational phrases with a critical boundary between them were used as the experimental stimuli. The roles of the three acoustic cues at the critical boundary were manipulated in five conditions. The first condition featured none of the acoustic cues. The following three conditions featured only one cue each: pause, final lengthening, and pitch reset, respectively. The fifth condition featured both pause duration and pre-final lengthening. A baseline condition was also included in which all three acoustic cues were preserved intact. Listeners were asked to detect the presence of the critical boundaries in Experiment 1 and judge the strength of the critical boundaries in Experiment 2. The results of both experiments showed that listeners used all three acoustic cues in the perception of prosodic boundaries. More importantly, these acoustic cues were weighted differently across the two experiments: Pause was a more powerful perceptual cue than both final lengthening and pitch reset, with the latter two cues perceptually equivalent; the effect of pause and the effects of the other two acoustic cues were not additive. These results suggest that the weighting of acoustic cues contributes significantly to the perceptual differences of intonational phrase boundary.

## Introduction

Spoken language is hierarchically structured into prosodic units divided by prosodic breaks. While researchers disagree on the number and definitions of prosodic units, they generally agree that prosodic units include prosodic words, phonological phrases, and intonational phrases [1]–[4]. There is a high correspondence of intonational phrase boundaries (IPBs) with major syntactic boundaries such as clause and sentence boundaries, which are central to language comprehension [5], [6]. Thus, the phrasing of intonational phrases has been the subject of numerous studies in the areas of speech production and perception [7]–[10]. In this paper, we will investigate how acoustic correlates are weighted in the perception of IPBs.

Previous studies have established three major acoustic correlates of IPBs: pause, final lengthening, and pitch reset [3], [6], [8], . Specifically, pauses are always found to accompany IPBs [17]–[19]. Furthermore, syllable durations are longer at the end of an intonational phrase than in the middle of it, a phenomenon known as final lengthening or pre-boundary lengthening [3], [20]–[23]. Finally, pitch tends to decline across the course of an utterance and reset to a higher value after an IPB boundary ([24]–[26]; for a review of the prosodic correlates of IPBs, please see [27]). These prosodic correlates have been found to be helpful for listeners in speech segmentation [5], [28], [29], and recent studies using Event-Related Potentials (ERPs) have shown that the perception of boundaries accompanied by these prosodic correlates elicited the Closure Positive Shift(CPS), a brain ERP component known to reflect the perception of prosodic boundary [30]–[32].

Although it is relatively clear that IPBs often coincide with prosodic parameters such as pause, final lengthening, and pitch reset, which help listeners interpret prosodic boundaries, it is still unclear how these three cues are weighted on the perceptual side. Studies on how listeners weight these cues in the perception of IPBs have been scarce, and most findings are based on studies involving only two of the three major acoustic correlates [29], [33]–[35]. Scott [29] tested the effects of pause and phrase-final lengthening by using syntactically ambiguous sentences such as “ Kate or Pat and Tony will come.” where the position of a phrase boundary after “ Kate” represented one meaning, and after “Pat” another meaning. She found that the duration of a pause alone or the combined duration of a pause and final stressed syllable lengthening could provide listeners with a cue to the location of a phrase boundary, even in the absence of a disambiguating pitch contour. Furthermore, the duration of a pause was perceptually equivalent to the same duration of final lengthening and an accompanying pause combined. Similarly, Shen [35] found that in perception, pause seemed to be a more important cue than phrase-final lengthening, since only when the duration of phrase-final syllables was increased to a certain length could it cue syntactic boundaries. Lin and Fon [33] moved one step further by showing that the roles of temporal cues in perception were weighted differently for different purposes: Final lengthening was more important for participants to detect boundaries, while pause duration was more responsible in cuing boundary sizes. These three studies are limited in that only temporal cues are investigated. In Streeter [34], the role of pitch change was compared to other parameters: amplitude and duration pattern. It was found that both pitch contour and duration pattern were reliably used as cues in parsing ambiguous algebraic expressions. Amplitude by comparison appeared to be a less important cue that was only effective in combination with appropriate values of duration.

The studies described above compared either the role of pause or the role of pitch change with that of final lengthening. Thus, there is, as yet, no clear picture of how these three correlates are weighted in the perception of IPBs. This issue has been partially resolved by Zhang [36], who tested the roles of pause, pre-boundary lengthening, and pitch in the perception of prosodic boundaries with expressions such as “turkey salad and coffee” (no-boundary condition) vs. “turkey, salad, and coffee” (boundary condition). She found that for Chinese listeners, pitch reset was weighted more heavily than pause and pre-boundary lengthening. However, it is possible that this finding may have resulted from her experimental design. In the production of her experimental materials, the distinction in pause duration ranged from zero ms in the non-boundary condition to over 300 ms in the boundary condition. However, in the perception task, the maximum pause duration was set at 80 ms in the boundary condition. This manipulation probably reduced the contribution of pause, making pitch reset a much more pronounced acoustic cue for Chinese listeners.

As described above, whereas numerous studies have explored the acoustic correlates of IPBs in production, only a relatively small number have focused on how the acoustic correlates are weighted in the perception of IPBs, and no clear picture of it has emerged yet. In this study, we present two experiments explicitly testing the roles of pause, final lengthening, and pitch reset in Chinese. In line with Lin and Fon [33], we not only tested how these acoustic cues contributed perceptually to the presence of an IPB, but also investigated how they contributed to the perceived boundary strength of an IPB. In Experiment 1, we explored whether listeners' performance in a boundary perception task remained the same or was degraded as a result of the loss of these correlates. In Experiment 2, we examined whether the perceived strength of an IPB was affected by the weighting of the correlates.

## Experiment 1

### 1.1 Materials

Forty-eight sentences that were originally used in Li and Yang [30] were used in this experiment. Each sentence consisted of two intonational phrases with an explicit IPB between them, which was the critical boundary for the present study. We chose these well-formed sentences to allow for a precise acoustic realization of the crucial IPBs. For instance, in example (1a) below, the two intonational phrases were “想保持领先” and “花时间进行练习非常重要.” Thus, there was an IPB between the two phrases. The pre-boundary syllable was “先(xian1),”and the post-boundary syllable was “花(hua1).” They were both marked in bold. The presence of the acoustic features of an IPB in these sentences was confirmed by a detailed acoustic analysis carried out in PRAAT. The sentences clearly revealed the three main prosodic boundary cues that were characteristic of IPBs at the crucial boundary position: pause, final lengthening, and pitch reset. Furthermore, perceptual data from ERPs clearly showed that CPS, a brain component marking speech segmentation, was observed for the crucial IPBs. For details regarding the acoustic parameters and the CPS data, please see Li and Yang [30].

(1a) [想/保持/领**先**/]_IPB1_ [**花**/时间/进行/练习/非常/重要/]_IPB2_.
  [Xiang3/bao3chi2/ling3**xian1**]_IPB1_ [**hua1**/shi2jian1/jin4xing2/lian4xi2/fei1chang2/bi4yao4]_IPB2._If/you/want to/keep/ahead/, taking/time/to do/exercises/is very/necessary.‘If you want to keep ahead, it is very necessary to take time to do exercises.’

The 48 sentences served as the baseline condition in which no cues were manipulated and all prosodic correlates were preserved intact. Out of these 48 sentences, we created another five conditions. The first condition was a no-cue condition, in which all three acoustic correlates were removed. The following three conditions featured only one cue each: pause, final lengthening, and pitch reset, respectively. For instance, in the second condition, only pause was preserved while the other two acoustic correlates were removed. Likewise, in the third condition, only final lengthening was preserved while the other two acoustic cues were removed. This manipulation allows us to isolate the acoustic correlates and directly examine their relevance to the perception of IPB. The fifth condition featured both pause and final lengthening. We manipulated this condition because some studies have found that the combination of pause and final lengthening was a good indicator of boundary size [22], [37]. These five manipulated conditions plus the baseline condition yielded altogether six conditions for the present study.

The crucial procedure for creating the manipulated conditions involved removing one or more of the acoustic features at the critical IPBs. For the removal of pause duration, the silent pauses at the critical boundary position were removed based on visual inspection in PRAAT. The removal of final lengthening and pitch reset was more complicated. Instead of neutralizing the values of final lengthening and pitch reset as has been done in previous studies [12], [34], we followed a procedure of exchanging acoustic features to circumvent the problem of determining a priori the neutral value of a specific acoustic feature [36]. Specifically, we exchanged the acoustic features of the words pronounced at the IPBs with the acoustic features of the same words that were not pronounced at a boundary position. This was realized by using another 48 sentences in which the pre- and post-boundary syllables did not span an IPB boundary, but only a syllable boundary (SB).

A SB exists between two syllables that form a word in Chinese. A word in Chinese is usually a bigram (two-syllable word). For instance, the word “鲜花” (‘flower’) is composed of two syllables: 鲜 and 花. Two syllables that are parts of a word are usually pronounced with a within-word syllable boundary [38]. Syllable boundaries in Mandarin Chinese are pronounced without distinct acoustic correlates and are often used as a no-boundary control condition in the study of prosodic hierarchies [30]. An IPB, however, often exists between two clauses that form a sentence or accompanies the end of a sentence [6]. IPBs are often pronounced with acoustic correlates such as pause, final lengthening, and pitch reset [11]–[16].

For example, (1a) had the SB counterpart (1b), shown below. In (1b), the pre-boundary syllable “鲜 (xian1)” and the post-boundary syllable “花 (hua1)” had exactly the same pronunciation and location as “先 (xian1)” and “花(hua1)” in the IPB sentences but were pronounced together as a word with only a within-word syllable boundary between them. Of particular importance is that these syllable-boundary sentences were only used for the acoustic-exchanging procedures to create the five manipulated conditions in the present study, but not for the perception experiments as reported below. The syllable-boundary sentences were also materials from Li and Yang [30].

(1b) [商店里的/**鲜花**/散发出/阵阵/浓郁的/芳香]_IPB1_.
  [Shang1 dian4 li3 de0/**xian1 hua1**/san4 fa1 chu1/zhen4 zhen4/nong2 yu4 de0/fang1xiang1]_IPB1_.In the store/the flowers/emit/bouts of/full-bodied/aroma.‘The flowers in the store emit bouts of full-bodied aroma.’

**Figure 1:**
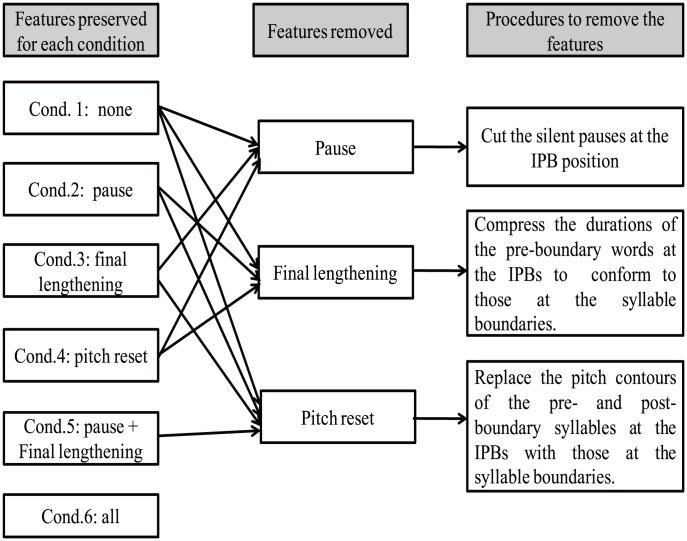
The procedures used to create the experimental conditions.

These SB counterparts were used in the procedures of exchanging acoustic parameters. For the removal of final lengthening at IPBs, the duration patterns of the pre-boundary syllables in the IPB sentences were compressed to conform to the duration patterns of pre-boundary syllables in the SB sentences. For the removal of pitch reset, the fundamental frequency contours for the pre- and post-boundary syllables from the SB sentences were superimposed on the pre- and post-boundary syllables in the IPB sentences. The procedures used to create the experimental conditions are shown in Fig. 1. The three major acoustic features of the critical boundaries in the five manipulated conditions as well as the baseline condition are shown in Table 1.

**Table 1:** Means of the acoustic parameters for the six conditions (with standard deviations in parentheses).

Conditions	Pause duration(s)	Syllable duration(s)	F0 reset (st)
No-cue	----	0.22 (0.05)	-0.42 (4.82)—
Pause	0.27 (0.11)	0.22 (0.05)	-0.42 (4.82)
Final lengthening	----	0.26 (0.04)	—0.42 (4.82)
Pitch reset	----	0.22 (0.05)	4.55 (4.68)
Pause + final lengthening	0.27 (0.11)	0.26 (0.04)	-0.42 (4.82)
Baseline	0.27 (0.11)	0.26 (0.04)	4.55 (4.68)

Note: Pause duration was measured as the duration of the silent interval at the IPBs. Final lengthening was measured as the duration of the pre-boundary syllable. Pitch reset was measured as the mean f0 differences between the two words before and after the boundaries. Pitch values were transformed into semitones through the following equation: St = 12 log_2_ (f0/f0_ref_). F0_ref_ was determined to be 70 Hz since the speaker for the experimental material was male in the present study [39].

Through the procedures described above, 48 sentence sets were created out of the original 48 sentences, with each set containing five manipulated sentences and one baseline sentence. Thus, altogether, 288 sentences were used for the perception experiment. The 48 sentence sets were counterbalanced according to a Latin square design and divided into six lists, with each sentence set presented only once within each list. Each list contained eight sentences per condition. To each list, 48 filler sentences with no sentence-internal IPB boundaries were also added. These filler sentences were added to balance the “Yes” and “No” responses of the task.

### 1.2 Ethics Statement

All participants provided written informed consent in accordance with the Declaration of Helsinki. The ethics committee of the Institute of Psychology, Chinese Academy of Sciences approved this study, including its participant recruitment procedure and methodology.

### 1.3 Participants

Twenty-four university students (13 women; mean age = 23.0 years; SD = 1.74) participated in the experiment for cash. All were native speakers of Chinese. All of them reported having no hearing problems.

### 1.4 Procedures and Data Analysis

The participants were tested individually in a sound-attenuating shielded chamber. They were seated in a comfortable chair approximately 60 cm in front of a monitor and were instructed to listen to the sentences attentively to detect speech boundaries. A trial started with a fixation cross, and 1000 ms later, a sentence was presented via headphones. At the end of each sentence, a question appeared on the screen which tested whether the participants had perceived the intended IPBs. For example, the question following sentence (1a) was “Do you perceive a boundary between “xian1” and “hua1”?” The participants were told to respond to this question by pressing “J” on the keyboard for a “Yes” response and “F” for a “No” response. The next trial began immediately after the participants gave their response. The experiment lasted about 15 minutes.

A repeated-measures analysis of variance (ANOVA) was performed with the factor condition as the independent variable and the mean proportions of boundaries detected by the participants as the dependent variable. Greenhouse-Geisser adjustment was used to correct for violations of sphericity. Post hoc comparisons were adjusted using Bonferroni's correction.

### 1.5 Results and Discussions

**Figure 2:**
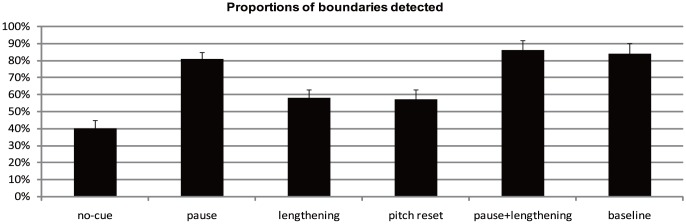
Proportions of the boundaries detected in the six experimental conditions (Error bars represent standard error of the mean).

Mean proportions of boundaries detected by the participants are displayed in Fig. 2. A repeated-measures ANOVA showed a main effect of condition, F (5, 115) = 18.47, *p*<0.001, *η* ^2^_partial_ = 0.45. Post hoc comparisons revealed that the proportion of boundaries detected for the no-cue condition was significantly lower than those for all the other five conditions (*ps*<0.05). The proportion of boundaries detected for the pause condition was significantly higher than those for the final lengthening *(p*<0.05) and pitch reset condition (*p*<0.05). Similarly, the proportions of boundaries detected for the pause + final lengthening condition were also significantly higher than those for the final lengthening *(p*<0.01) and pitch reset condition (*p*<0.01). This pattern was the same for the baseline condition: A higher proportion was found for the baseline condition than for the final lengthening *(p*<0.05) and pitch reset condition (*p*<0.05). There was no significant difference between the pause, pause + final lengthening, and baseline conditions *(ps*>0.05). The final lengthening and pitch reset conditions did not differ from each other *(p*>0.05).

The above results showed that participants' responses for boundary detection varied among the experimental conditions. However, note that except for the baseline condition, the five manipulated conditions were not natural speech but synthesized speech created in PRAAT. This could result in different degrees of naturalness, which may confound the effects of the acoustic parameters on the participants' responses for boundary detection. To examine whether the perceived boundary strength was influenced by the degrees of naturalness, we conducted a posttest by asking another 24 participants to rate the naturalness of the sentences on a scale of 1 (very unnatural) to 7 (very natural). The results for the naturalness rating are given in Table 2.

**Table 2:** Naturalness rating scores for the six conditions.

Conditions	Means	Standard deviations
No-cue	3.49	1.23
Pause	5.04	0.77
Final lengthening	4.12	0.97
Pitch reset	4.17	1.03
Pause + final lengthening	5.20	0.63
Baseline	5.45	0.81

**Figure 3:**
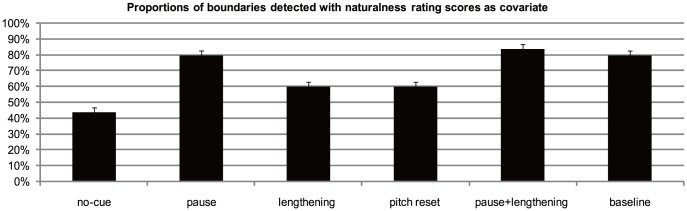
Proportions of the boundaries detected with naturalness rating as covariate.

A repeated-measures ANOVA for the rating scores showed a main effect of condition, F (5, 115) = 25.83, *p*<0.001, *η* ^2^_partial_ = 0.53. Post hoc comparisons revealed that the no-cue condition was rated as less natural than all the other five conditions (*ps*<0.05). Furthermore, the pause condition was rated as more natural than the lengthening condition (*p*<0.01) and pitch reset condition (*p*<0.05). The pause + final lengthening condition was also rated as more natural than the lengthening condition (*p*<0.001) and pitch reset condition (*p*<0.01). Rating scores were higher for the baseline condition than for the lengthening condition (*p*<0.01) and pitch reset condition (*p*<0.01). No other differences were significant (*ps*>0.05). Thus, it appears that the naturalness of the sentences differed across conditions. To control for the influence of naturalness on the results of boundary detection, we ran a univariate analysis with condition as the independent variable, naturalness rating score as the covariate, and mean proportion of boundaries detected as the dependent variable. The results are shown in Fig. 3.

As shown in Fig. 3, when the influence of naturalness degree was controlled for, the resulting pattern was almost identical to that shown in Fig. 2. This impression was confirmed by statistical analysis. The results of the univariate analysis revealed a main effect of condition, F (5, 281) = 16.60, *p*<0.001, *η* ^2^_partial_ = 0.23. Post hoc comparisons revealed that the proportion of boundaries detected was significantly lower in the no-cue condition than in all the other five conditions (*ps*<0.01). The proportion of boundaries detected for the pause condition was significantly higher than those for the final lengthening condition (*p*<0.01) and pitch reset condition (*p*<0.01). Similarly, the proportion of boundaries detected for the pause + final lengthening condition was significantly higher than those for the final lengthening condition (*p*<0.01) and pitch reset condition (*p*<0.01). Finally, the proportion was also higher for the baseline condition than for the final lengthening condition (*p*<0.01) and pitch reset condition (*p*<0.01). No significant differences were found between the pause, pause + final lengthening, and baseline conditions (*ps*>0.05). There was also no significant difference between the final lengthening and pitch reset conditions (*p*>0.05). These results suggest that naturalness degree was not a confounding factor for the results of boundary detection.

The above results showed that acoustic cues were weighted differently in the detection of IPBs: Pause was the strongest indicator of an IPB; final lengthening was perceptually equivalent to pitch reset; the effect of a pause and the effects of the other two acoustic cues were not additive. However, these results alone cannot provide us with a full picture of how the three acoustic cues are weighted in the perception of IPBs. It has been shown that listeners are sensitive not only to the presence or absence of a boundary, but also to how strong a boundary is [8], [33]. We believe that listeners' weighting of acoustic cues is not only displayed in their judgment of boundary presence; instead, perceptual sensitivity to acoustic cues should also be reflected in boundary strength judgments, as has been shown in previous research [33]. Therefore, in Experiment 2, we address the roles of the three acoustic parameters in the perceived strength of an IPB.

## Experiment 2

### 2.1 Materials

The materials of Experiment 1 were used.

### 2.2 Ethics Statement

All participants provided written informed consent in accordance with the Declaration of Helsinki. The ethics committee of the Institute of Psychology, Chinese Academy of Sciences approved this study, including its participant recruitment procedure and methodology.

### 2.3 Participants

Twenty-four undergraduate students (12 women; mean age = 22.3 years; SD = 2.61) participated for financial compensation. All were native speakers of Chinese with normal hearing. None of them had participated in Experiment 1.

### 2.4 Procedures and Data Analysis

The procedures were the same as those of Experiment 1 except that the participants were asked to perform a different task in this experiment. Instead of judging the absence or presence of a boundary, they were asked to indicate how strong a boundary was on a 7-point scale from 1 “no boundary at all” to 7 “a very strong boundary.” For example, the question following sentence (1a) was “How strong is the boundary between “xian1” and “hua1?” The participants gave their answers by pressing the appropriate number keys on the keyboard.

A repeated-measures ANOVA was performed with the factor condition as the independent variable and perceived boundary strength as the dependent variable. Greenhouse-Geisser adjustment was used to correct for violations of sphericity. Post hoc comparisons were adjusted using Bonferroni's correction.

### 2.5 Results

**Figure 4:**
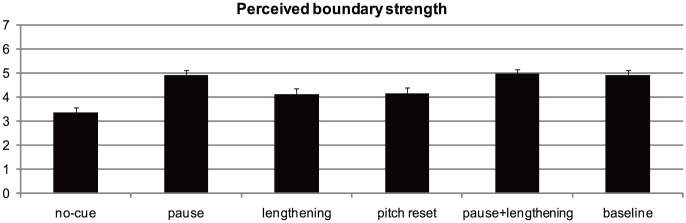
Perceived boundary strength across the six experimental conditions.

As shown in Fig. 4, participants perceived stronger boundaries in the pause, pause + final lengthening, and baseline conditions than in the no-cue, final lengthening, and pitch reset conditions. This indicates that perceived boundary strength was stronger when a pause was present as opposed to absent. This impression was confirmed by statistical analysis. A repeated-measures ANOVA showed a main effect of condition, F (5,115) = 15.39 *p*<0.001, *η* ^2^_partial_ = 0.40. Post hoc comparisons revealed that the perceived strength for the no-cue condition was significantly weaker than for all the other five conditions (*ps*<0.05). Perceived strength for the pause condition was significantly stronger than that for the final lengthening condition (*p*<0.05) and pitch reset condition (*p* = 0.05). Stronger boundary strength was also found for the pause + final lengthening condition than for the final lengthening condition (*p*<0.01) and pitch reset condition (*p*<0.05). Finally, perceived strength was also stronger for the baseline condition than for the final lengthening condition (*p* = 0.06) and pitch reset condition (*p* = 0.07). There was no significant difference between the pause, pause + final lengthening, and baseline conditions (*ps*>0.05). The final lengthening and pitch reset conditions did not differ from each other (*p*>0.05).

**Figure 5:**
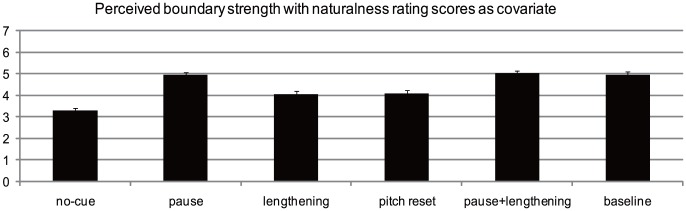
Perceived boundary strength with naturalness rating as covariate.

These results showed that perceived boundary strength varied among experimental conditions. As with Experiment 1, to test whether the influence of naturalness degree was a confounding factor for the results of perceived boundary strength, we ran a univariate analysis with condition as the independent variable, naturalness rating score as the covariate, and perceived boundary strength as the dependent variable. The results (shown in Fig. 5) again showed a main effect of condition, F (5,281) = 18.97, *p*<0.001, *η* ^2^_partial_ = 0.25. Post hoc comparisons revealed that the no-cue condition was perceived as less strong than all the other five conditions (*ps*<0.001). Perceived strength for the pause condition was significantly stronger than that for the final lengthening condition (*p*<0.001) and pitch reset condition (*p*<0.001). Stronger boundary strength was also found for the pause + final lengthening condition than for the final lengthening condition (*p*<0.001) and pitch reset condition (*p*<0.001). Finally, perceived strength was also stronger for the baseline condition than for the final lengthening condition (*p*<0.001) and pitch reset condition (*p*<0.001). No significant difference was found between the pause, pause + final lengthening, and baseline conditions (*ps*>0.05). There was also no significant difference between the final lengthening and pitch reset conditions (*p*>0.05). These results suggest that naturalness degree was not a confounding factor in our results. Thus, a strong pattern emerged whereby pause appeared to be a more powerful perceptual cue for the perceived strength of an IPB than final lengthening and pitch reset, with the latter two cues perceptually equivalent.

## General Discussion

The aim of the present study was to test listeners' weighting of acoustic cues in their perception of IPB. The roles of three acoustic cues (pause, final lengthening, and pitch reset) were examined in two experiments. In Experiment 1, we examined how listeners weighted these acoustic cues in the detection of prosodic boundaries, while in Experiment 2, we examined how they weighted the same acoustic cues in the perceived strength of prosodic boundaries. The results of the two experiments consistently showed that the three acoustic cues played significant roles in the perception of IPB. More importantly, we found that they were weighted differently: Of the three cues, listeners relied most heavily on pause. Final lengthening and pitch reset were not heavily weighted, and these two cues were perceptually equivalent. Finally, the effect of pause and the effects of the other two acoustic parameters were not additive. These results suggest that acoustic cues are weighted differently not only in the detection of boundary presence, but also in the judgment of boundary strength.

In Experiment 1, we found that the proportions of boundaries detected were significantly lower in conditions where none of the acoustic cues were present than in conditions where one or more of the acoustic cues were present. This suggests an important role of the acoustic cues in speech segmentation. Note that for the no-cue condition, the proportions of prosodic boundaries detected were around 40%. This might be because we used syntactically unambiguous sentences as the experimental material, and the explicit syntactic structure of the sentences probably has a predictive power on where a break is placed, as has been found previously [40]. More importantly, in Experiment 1, we found that the three acoustic cues were weighted differently for the detection of an IPB. Proportions of boundaries detected in conditions where only pause was present (81%) were significantly higher than in conditions where only final lengthening (58%) or pitch reset (57%) was present. This suggests that boundary pause was perceptually more powerful than both final lengthening and pitch reset, while the latter two acoustic cues were perceptually equivalent in terms of the perceptual effects on listeners.

In Experiment 2, we found that the no-cue condition was less natural than all the other five conditions. This suggests that IPBs were perceived as more natural when they were accompanied by acoustic cues. Consistent with Experiment 1, Experiment 2 revealed the same pattern of acoustic weighting in the perceived strength of IPBs: Pause appeared to be a more powerful perceptual cue for the perceived strength of an IPB than final lengthening and pitch reset, with the latter two cues perceptually equivalent. These results suggest that listeners' weighting of acoustic cues is not only displayed in their judgment of boundary presence, but also in their judgment of boundary strength.Previous research has found that pause is more responsible in cueing boundary size than final lengthening [33]. Our results extend this finding by showing that pause is more heavily weighted in cuing boundary size than both final lengthening and pitch reset. The fact that listeners can perceive boundaries of the same prosodic category to be of different strengths has already been noted by prior studies [3], [9]. Our results add to the literature by showing that listeners can perceive IPBs to be of different strengths simply based on the acoustic cues presented to them.

The results of both experiments showed that the pause condition was perceptually equivalent to the pause + final lengthening and baseline conditions. This suggests that perception of an IPB is heavily dependent on the presence of pauses, even to the extent that it may overrule the contribution of other parameters such as pre-boundary lengthening and pitch reset. The perceptual equivalence of a grammatical pause and a pause + final lengthening has been noted in Scott [29]. Our results extend this finding by indicating a perceptual equivalence between pause and pause plus the other two acoustic parameters (final lengthening and pitch reset).

Across both experiments, no significant difference was found between final lengthening and pitch reset in terms of their perceptual effects on the listeners. This implies a discrepancy between production and perception: In the production of an IPB in Chinese, pitch reset was found to be a more reliable cue than final lengthening [41]; however, in perception, we found that pitch reset was only perceptually equivalent to final lengthening. This may have occurred because Chinese is a tonal language, and listeners who speak a tonal language are more sensitive to lexical tones but less sensitive to F0 information at the sentence level [42]. Thus, although in production pitch reset varies more dramatically than final lengthening, for Chinese listeners, these two cues can be perceptually equivalent.

One thing that should be noted is that although we only used IPBs in the present study, it is difficult to know whether an IPB is still perceived as one when cues are removed or manipulated. In studies that have manipulated acoustic cues at IPBs, listeners showed the closure positive shift, a particular ERP component known to reflect the perception of IPBs, independent of the presence of a pause cue, suggesting that the remaining cues in combination were sufficient for successful perception of IPBs [30]–[32]. In the present study, we found that the presence of a pause was a powerful indicator of an IPB: The proportions of boundaries detected in the pause condition (81%) did not significantly differ from those in the baseline condition where all cues were present (84%). This suggests that the removal of final lengthening and pitch reset did not affect the perception of IPBs. However, the presence of a pitch reset or final lengthening was not a strong indicator of an IPB: The proportions of boundaries detected in the pitch reset condition (57%) and in the final lengthening condition (58%) were quite low. This suggests that the removal of pause plus another primary cue (final lengthening or pitch reset) could significantly affect the perception of IPBs. Therefore, it appears that whether an IPB is still perceived as one depends on which cues are present.

It should be noted that one reason listeners might be more affected by the removal of one cue compared to another may have to do with the salience of these variations for listeners. Previous study has suggested that only when the salience of two acoustic cues is comparable can their relative contributions be assessed [43]. In the present study, due to the acoustic-feature exchanging technique that we used to create the experimental materials, we could not equate the salience of the acoustic cues before their contributions were assessed. However, acoustic salience alone could not explain our results. As reflected by our acoustic analysis in Table 1, compared to the values of pitch reset and final lengthening in the no-cue condition, the value of resetting in the pitch reset condition was 4.13 St larger, and the value of lengthening in the final lengthening condition was 40 ms larger. Given that 5St and 100 ms are perceptually comparable [43], it can be argued that the extent of pitch resetting was greater than that of final lengthening in production for our experimental materials. However, across the two experiments, the conditions of final lengthening and pitch reset were perceptually equivalent. Thus, it is unlikely that our results were due to the differences of acoustic salience. Nonetheless, future studies will need to explore whether changes in acoustic cues modulate phrase perception when the salience of the acoustic cues is experimentally equated, which would require a pre-test to determine how the salience of a change in one cue can be equated with that of other cues.

## Conclusions

In summary, the contribution of this research is that it is the first study to systematically manipulate the three acoustic correlates to examine cue weighting in the perception of IPBs. It is clear from the results that acoustic cues were weighted differently for the perception of IPBs: Pause was a more powerful perceptual cue than both final lengthening and pitch reset, with the latter two cues perceptually equivalent. Also, the effect of pause and the effects of the other two acoustic cues were not additive. However, this study is limited in that we only studied acoustic weighting in Chinese. Given that prosodic features vary across languages, which can result in different patterns of cue weighting across languages, future studies are needed to compare the perceptual weighting of the prosodic cues in different languages. Furthermore, note that we chose natural speech with well-formed structures to allow for a more natural acoustic realization of the boundaries. This is important in that it resembles what we encounter in everyday life. However, this could have reduced the effect of acoustic features in cueing boundaries. Thus, future studies are also needed to systematically explore the weighting of the three major acoustic correlates in syntactically ambiguous sentences.
